# Preliminary Results of Microsurgical Sperm Retrieval in Azoospermic Patients: A Randomized Controlled Trial Comparing Operating Microscope vs. Surgical Loupes

**DOI:** 10.3390/jcm14030970

**Published:** 2025-02-03

**Authors:** Mirko Preto, Luca Boeri, Lorenzo Cirigliano, Marco Falcone, Valentina Parolin, Federica Peretti, Ilaria Ferro, Natalia Plamadeala, Martina Scavone, Emanuele Zupo, Paolo Gontero

**Affiliations:** 1 Urology Clinic, A.O.U. “Città della Salute e della Scienza”, Molinette Hospital, University of Turin, 10126 Torino, Italy; mirko.preto@unito.it (M.P.); dr.lorenzocirigliano@gmail.com (L.C.); ilariaferro@unito.it (I.F.); nataliaplamadeala@unito.it (N.P.); martinascavone@unito.it (M.S.); emanuelezupo@unito.it (E.Z.); paologontero@unito.it (P.G.); 2 IRCCS Fondazione Ca’ Granda, Ospedale Maggiore Policlinico, 20122 Milan, Italy; luca.boeri@gmail.com (L.B.); valentinaparolin@gmali.com (V.P.); 3 Neurourology Clinic, A.O.U. “Città della Salute e della Scienza”, Unità Spinale Unipolare, 10126 Turin, Italy; 4 Department of Surgical Sciences-Urology, Città della Salute e della Scienza di Torino, University of Turin, 10126 Torino, Italy; 5 Department of Urology, School of Medicine, Biruni University, 34015 Istanbul, Turkey

**Keywords:** TeSE, male infertility, surgical loupes, cryopreservation, micro-TeSE, non-obstructive azoospermia

## Abstract

**Objectives:** To compare surgical outcomes and sperm retrieval rates (SRRs) between conventional microsurgical-assisted testicular sperm extraction (m-TeSE—Group A) and testicular sperm extraction performed with surgical loupes (l-TeSE—Group B) in adult males with non-obstructive azoospermia (NOA). **Methods:** A multicentric prospective randomized trial (ethics committee no. 202/2022) in accordance with the CONSORT guidelines was conducted from March 2022 to April 2024. Adult males with NOA without genetic alterations who signed the informed consent were enrolled. SRRs, intra- and postoperative complications (according to the Clavien–Dindo classification), and hormonal profile changes were considered as outcomes during the follow-up period. **Results:** A total of 42 NOA patients were enrolled. The median age was 35 years (IQR: 33–49). The preoperative median FSH was 16.5 mIU/mL (IQR: 11.6–22.5) and the total testosterone was 4.6 (3.5–5.6). Overall, the SRR was 22.6%, with sperm retrieved from 19 testes. Histopathological findings reported Sertoli cell-only syndrome (SCOS) in 46.4% (39 cases), hypospermatogenesis in 26.2%, and germ cell arrest in 26.2% of the patients. No intraoperative complications were recorded. The postoperative complications were minimal (Clavien–Dindo grade I), but no significant differences were recorded in-between the two surgical approaches. Considering the operative time of the testicular exploration alone, Group B seemed to be faster than the m-TeSE, with a median time saving of 8 min (*p* < 0.01). **Conclusion:** The use of surgical loupes was safe and comparable with m-TeSE in terms of the SRRs and complication rates. L-TeSE offered a reduction in the operative time compared with m-TeSE.

## 1. Introduction

Azoospermia, defined as the complete absence of sperm in the ejaculate, accounts for up to 20% of male infertility cases, with non-obstructive azoospermia (NOA) being significantly more prevalent than obstructive azoospermia (OA). NOA presents a significant challenge for andrologists due to idiopathic failures in spermatogenesis [1,2]. Prior to the 1990s, couples facing NOA had limited reproductive options, primarily relying on adoption or donor sperm. However, the landscape has dramatically evolved with pivotal advancements in reproductive medicine.

The advent of intracytoplasmic sperm injection (ICSI) marked a groundbreaking shift, enabling successful pregnancies with minimal sperm quantities [3]. Furthermore, the discovery that sperm could be retrieved directly from the testes for in vitro fertilization (IVF) and ICSI opened new avenues for couples affected by NOA [4]. It became evident that even men with severe testicular failure might harbor localized spermatogenic foci, from which sperm can be surgically retrieved for ICSI, leading to live births. This realization spurred the development of advanced surgical techniques aimed at optimizing sperm retrieval in NOA patients.

Conventional testicular sperm extraction (TeSE), first described in 1993, involves an incision in the tunica albuginea and the surgical dissection of seminiferous tubules, with the aim of locating sperm via blind random sampling [5]. While TeSE has provided a framework for sperm retrieval, its inability to accurately identify the foci of normal sperm production has led to the development of more refined techniques, such as trifocal TeSE and microsurgical-assisted TeSE (m-TeSE) [6,7]. 

Traditionally, sperm extraction performed using an operative microscope with 20–36× magnification was considered the gold standard for m-TeSE in NOA patients. However, recent studies suggest that surgical loupes, offering a 3.5–5× magnification, provide comparable outcomes to the operating microscope in various surgical fields, including urology [8,9,10]. The advantages of loupe magnification include shorter operative times, reduced costs, and the potential to perform microsurgery in settings where an operating microscope is unavailable.

This study aimed to directly compare the efficacy of these two optical magnification tools (m-TeSE versus l-TeSE) by evaluating the SRRs and surgical outcomes in a prospective randomized controlled trial.

## 2. Materials and Methods

This randomized multi-center controlled trial was approved by the ethics committee of Città della Salute e della Scienza di Torino, Italy (protocol no. 202/2022). This study included adult males diagnosed with NOA who required an m-TeSE. Azoospermia was preoperatively confirmed twice through centrifuged semen pellet analysis.

The exclusion criteria were as follows:

- Absence of signed written informed consent;
- Age < 18 years;
- Obstructive azoospermia;
- Genetic anomalies (e.g., Klinefelter syndrome, Kallmann syndrome, Y chromosome microdeletions, CFTR mutations);
- Previous testicular biopsies/surgical sperm retrieval;
- Personal history of malignant testicular tumor;
- Unilateral cryptorchidism;
- Varicocele;
- Previous chemotherapy/radiotherapy treatments;
- Monorchidism.

We excluded patients with conditions such as unilateral disease processes due to the potential bias in the success of sperm retrieval in one testis but not the other.

Sperm extraction procedures were performed using an operative microscope (20–36× magnification) on one testicle (Group A—m-TeSE) and surgical loupes (3.5–5×) on the other testicle (Group B—l-TeSE). Randomization was determined via a computerized random selection generator on the day of the procedure, without taking into account any biological parameters other than the right or left side and the type of surgical technique to select which magnification tool should be used on each testicle.

The testicular volume was measured preoperatively using a standardized Prader’s orchidometer. The surgical outcomes, including the operative time, hospital stay, SRRs, and postoperative complications (defined according to the Clavien–Dindo classification [11]), were recorded. The hormonal levels were evaluated preoperatively and six months postoperatively. The histopathology of the testicular biopsies were described with both the Johnsen score and the McLachlan et al. [12] classification.

Spermatogenesis on the testicular biopsies was thus classified as follows:

- *Normal testicular biopsy*: full spermatogenesis in the entire biopsy with the presence of a normal inter-tubular tissue.
- *Hypospermatogenesis*: All phases of spermatogenesis are present, though diminished to differing extents. This definition also encompasses diverse patterns, where some tubules may display an epithelium composed solely of Sertoli cells.
- *Germ cell arrest*: This refers to a complete arrest at a specific stage of spermatogenesis, most frequently at the spermatogonial or primary spermatocyte stage. Arrest during spermiogenesis is relatively rare. In cases where a small number of spermatids are detected in a single tubule, while the remaining tubules contain only primary spermatocytes, the condition should not be labeled as germ cell arrest. Instead, it should be categorized as severe hypospermatogenesis.
- *Sertoli cell-only syndrome*: This term is applied exclusively in cases where there is a complete absence of germ cells within all seminiferous tubules, and the tubules contain only Sertoli cells. In such instances, there is no evidence of spermatogenesis at any stage, and the seminiferous epithelium is composed solely of Sertoli cells without any other cell types involved in the sperm production process.
- *Seminiferous tubule hyalinization:* this describes the presence of tubules that lack both germ cells and Sertoli cells, and is typically associated with peritubular fibrosis and the buildup of material resembling a basement membrane in the peritubular region.
- *Carcinoma* in situ *(CIS):* this term is used for pre-invasive malignant CIS cells, which are usually present in the place normally occupied by spermatogonia.
- *Immature testis:* This pattern is uncommon in adult infertile men but is characteristic of hypogonadotrophic hypogonadism. The seminiferous epithelium contains immature Sertoli cells along with germ cells, typically gonocytes or spermatogonia, while the tubules lack a defined lumen. Additionally, the interstitial space either lacks Leydig cells or contains very few identifiable ones.

### 2.1. Statistical Analysis

The normality of the variable distributions was tested by the Kolmogorov–Smirnov test. The categorical variables were described using frequencies and percentages. For the continuous variables with normal distributions, the means and standard deviations (SDs) were reported, whereas variables with non-normal distributions were described using medians and interquartile ranges (IQRs). The differences between groups were assessed by the chi-square test or Fisher’s exact test for discrete variables, depending on the expected cell counts. For the continuous variables, differences between groups were assessed by Student’s *t*-test for the normally distributed variables and the Mann–Whitney U test or the Wilcoxon test (for independent and paired samples, respectively) for the non-normally distributed variables. A *p*-value < 0.05 was considered statistically significant. The statistical analyses were performed using the Statistical Package for the Social Sciences (SPSS v. 28; IBM, Chicago, IL, USA).

### 2.2. Surgical Technique

All patients underwent antibiotic prophylaxis with a third-generation cephalosporin. For patients allergic to cephalosporins, prophylaxis with an aminoglycoside was used. The procedure was performed with the patient in the supine position, under spinal or general anesthesia, depending on the patient’s characteristics and needs. The sterile surgical field was prepared, exposing only the scrotum to reduce possible microbial contamination. A longitudinal incision of approximately 3 cm was made along the scrotal raphe using a scalpel. Surgical dissection proceeded layer by layer until access to one hemiscrotum was obtained. The testis was separated from the surrounding dartos and luxated outside the scrotal sac. At this point, a longitudinal incision was made in the tunica vaginalis to allow for complete exposure of the tunica albuginea. Two Vicryl 2-0 traction sutures were placed 0.5 cm cranially and caudally to the testicular equatorial line, which was subsequently incised with a scalpel for 3/4 of the testicular circumference. Using the previously placed traction sutures, the testicular parenchyma was widely exposed.

**Figure 1:**
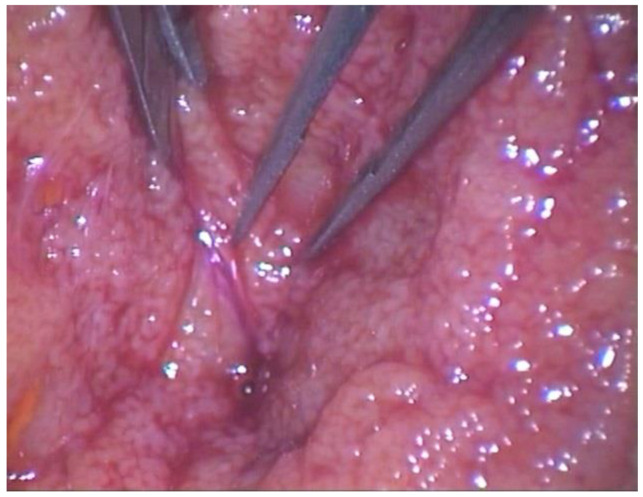
Through the operative microscope, the surgeon searched for the more dilated, more opaque, and whiter seminiferous tubules, possibly located near the blood vessels.

In the testes selected for M-TESE, through the operating microscope, a direct examination of the testicular parenchyma was performed under magnification (20–25×) (Figure 1). The surgeon searched for the more dilated, more opaque, and whiter seminiferous tubules, possibly located near the blood vessels. Multiple testicular specimens were excised. To explore both the superficial and deep testicular lobes, dissection was performed with microsurgical forceps, aiming to preserve the blood supply. One or more specimens were taken for histopathological examination. In cases of low-retrieval-chance NOA, characterized by a low testicular volume (<10 mL) and high FSH levels (>12.4 IU/L), the patients underwent a combined trifocal and microsurgical approach to enhance the sperm retrieval rate, as observed by Falcone et al. [6]. The tunica albuginea was then closed using an adsorbable Vicryl 4-0 continuous suture, while the tunica vaginalis was subsequently sutured with adsorbable Vicryl 3-0. After a thorough hemostasis check, the testis was repositioned in the corresponding hemiscrotum, ensuring that the spermatic cord was not twisted. The same procedure was performed on the contralateral side, where instead of the operating microscope, the surgeon used surgical loupes for magnification (3.5–5×). Absorbable sutures were applied for the dartos and skin layers. A Foley catheter was routinary placed and removed after 6 h or the next day. A non-compressive dressing was placed over the surgical wound, and ice was applied to reduce the pain.

## 3. Results

From March 2022 to April 2024, a total of 42 NOA patients who met the inclusion criteria were enrolled in this study and underwent magnified sperm extraction. The median age of the participants was 35 years (IQR: 33–49). Among them, 19 patients (45.2%) were active smokers, and 2 patients (4.8%) had type I diabetes. The preoperative follicle-stimulating hormone (FSH) level was 16.5 mIU/mL (IQR: 11.6–22.5), while the luteinizing hormone (LH) and testosterone were, respectively, 7.4 mIU/mL (IQR: 5.2–10.1) and 4.6 ng/mL (IQR: 3.5–5.6). The median testicular volumes were 10.0 cc (IQR: 8.0–14.2) on the left and 10.0 cc (IQR: 8.0–14.0) on the right side.

The surgical outcomes are reported in Table 1. The median operative time was 33 min (IQR: 31–38) for Group A and 25 min (IQR: 24–27) for Group B, with a statistically significant difference between the two groups (*p* < 0.01). The overall SRR was 22.6%, with no significant difference between Group A and Group B (21.4% vs. 23.8%, *p* = 0.79). The histopathological evaluations and Johnsen scores revealed no significant differences between the groups. Sertoli cell-only syndrome (SCOS) was identified in 39 testes (46.4%), germ cell arrest (GCA) in 22 (26.2%), and hypospermatogenesis (HS) in 20 (23.8%), while only 3 testes (3.6%) exhibited normal spermatogenesis. The median Johnsen score was 3.0 (IQR: 2.0–3.4), with no significant difference between groups. No significant intraoperative or postoperative complications (greater than grade 1 according to Clavien–Dindo) were observed. Only one case of postoperative hematoma, which was resolved spontaneously, was noted in Group A (1.2%—Clavien–Dindo grade 1). No significant differences were found in the postoperative complications overall.

While no cases of postoperative hypogonadism were reported, and although the values remained within the reference range, there was a significant reduction in the total testosterone levels at the six-month follow-up (see Table 2).

## 4. Discussion

Non-obstructive azoospermia (NOA) is a complex condition defined by the absence of sperm in the ejaculate, confirmed through two separate semen analyses. It accounts for a significant portion of male infertility cases, affecting around 10–15% of infertile men and about 1% of the general male population [1]. Unlike obstructive azoospermia, NOA results from a failure in sperm production. Men with NOA typically exhibit elevated follicle-stimulating hormone (FSH) levels (>7.6 IU/mL), and some may have low total testosterone levels and reduced testicular volume. The causes of NOA are diverse, accounting for 60–70% of azoospermia cases. Contributing factors include a history of cryptorchidism; testicular torsion or trauma; infections, like mumps orchitis; toxin exposure (e.g., chemotherapy or radiotherapy); and genetic disorders, such as Klinefelter syndrome and Y chromosome microdeletions. However, in nearly half of NOA cases, the underlying cause of sperm production failure remains unknown [13].

NOA is thus a major cause of male azoospermia and represents one of the primary challenges in andrology, as it mainly results from failed spermatogenesis, with areas of spermatogenesis often scattered focally within the testicular tissue. Current evidence suggests several surgical options for NOA patients, including fine needle aspiration (FNA), testicular sperm aspiration (TESA), TESE, and microsurgical-assisted TESE (M-TESE):

- **FNA** does not yield viable sperm but is used to map the testis for focal spermatogenesis. If spermatogenesis is widespread, TESA or TESE may be suitable, while M-TESE is preferred for rare focal areas. Since FNA often requires further surgery and may damage the testis, it is not recommended for NOA patients [14].
- **TESA** involves extracting testicular tissue using a biopsy needle. While safe and cost-effective, it often leads to additional procedures. TESE was shown to double the sperm retrieval rate (SRR) compared with TESA, making TESA less advisable for NOA patients [15].
- **TESE** revolutionized NOA treatment in 1993 when it enabled successful sperm retrieval for ICSI [16]. Conventional TESE (c-TeSE), often described as “random,” lacks the ability to selectively target seminiferous tubules that are more likely to contain sperm. Moreover, c-TeSE carries the risk of complications, such as temporary or permanent testosterone reduction due to testicular devascularization, emphasizing the need for more refined techniques [17,18]. While early results showed up to 50% of patients with NOA had viable sperm in the c-TESE procedure, the trifocal TESE approach now offers better outcomes due to the uneven distribution of spermatogenesis in NOA patients. A single biopsy may not capture areas of normal spermatogenesis. Studies, like the one from Ostad et al. [19], confirmed that trifocal TESE is more effective than unifocal TESE, though concerns exist about its potential impact on testicular endocrine function, despite recent analyses that showed no significant hypogonadism after 24 months [18].
- **M-TESE**, developed in 1999, uses optical magnification (20–25×) to identify larger, opaque seminiferous tubules, which are more likely to contain spermatozoa [6,20,21], which is particularly useful for NOA patients where sperm production is focal. M-TESE demonstrated excellent sperm retrieval rates with fewer complications compared with conventional TESE [22]. However, the adoption of m-TeSE is limited by its high cost and the need for specialized microsurgical training [7].

Some recent studies suggest that alternative or combined techniques, such as using trifocal TeSE and m-TeSE, could further improve the retrieval rates, particularly in NOA cases with a low success probability [6]. This technique was also applied in our study, performing the combined trifocal M-TESE in so-called “low chances” retrieval NOA patients, identified by a low testicular volume (<10 mL) and high FSH levels (>12.4 IU/L). Predictive factors for sperm retrieval success include patient age, clinical history, hormonal profiles, testicular volume, and histology [7,23,24,25]. While the relationship between the testicular volume and spermatogenesis is debated, histological categories were shown to correlate strongly with sperm retrieval outcomes [7,25,26]. 

With the advancements in surgical techniques, m-TeSE has emerged as the gold standard, with sperm retrieval success rates ranging from 15% to 50% in NOA cases [5,15,27]. However, the microsurgical complexity of m-TeSE (requiring an operating microscope, specialized instruments, and highly skilled surgeons) can limit its accessibility in many centers [5]. Microsurgery emerged from the combination of technological advancements and clinical demand. The operating microscope was first introduced in 1921 by Carl Olof Nylen, a surgical intern in Sweden, to assist with middle ear surgeries for otosclerosis. His modification of a monocular dissecting microscope, later improved by Gunnar Holmgren, marked its first use in otolaryngology. In 1946, Richard Perritt adapted a binocular microscope for ophthalmology, revolutionizing cataract surgery. The Zeiss Company (Oberkochen, Germany) then developed advanced microscopes for gynecologic surgery, with Kurt Swolin using them in 1967 to correct fallopian tube obstructions. This led to the widespread adoption of microsurgery across specialties. Urology first utilized microsurgery for vasal and epididymal obstructions, with early vasovasostomies reported in 1977 by Drs. Owen and Silber. Microsurgery soon expanded into other urological fields, including vascular surgery, tissue grafting, and robotics [28]. In 1999, Schlegel [22] proposed the use of microsurgery in assisted reproduction, and since then, M-TESE has become a fundamental technique for sperm retrieval worldwide.

In this study, we compared the SRR of conventional m-TeSE with a less demanding procedure based on the use of surgical loupes (l-TeSE) in non-genetic azoospermic patients. Our results show that the l-TeSE was non-inferior to the m-TeSE in terms of the retrieval rate. Additionally, we observed some significant differences between the two approaches in the following areas:

- Reduction in operative time: One of the main advantages of l-TeSE was the reduced operative time. Although surgical loupes provide lower magnification than an operating microscope (3.5–5× vs. 20×, respectively), this was proven sufficient to identify and retrieve areas of residual spermatogenesis. This technical simplification led to shorter operative times, which enhanced the overall surgical workflow. In different surgical fields, some series have demonstrated the use of surgical loupes to perform procedures previously carried out with an operating microscope, showing comparable outcomes in terms of the results and complications [9,10]. This further supports the adoption of l-TeSE in specific scenarios.
- Comparable surgical complications: In terms of safety, m-TeSE is known for its low complication rate, consistent with the current knowledge and best practices in surgical management. Using the Dindo–Clavien classification [11], we found that the surgical complication rates were comparable between the m-TeSE and l-TeSE, with no significant differences. This suggests that l-TeSE is equally safe. Furthermore, the lower magnification in the l-TeSE did not seem to negatively impact the safety outcomes compared with the m-TeSE. Considering postoperative hormonal changes, the follow-up period was limited to six months. Although no cases of hypogonadism were reported, a significant reduction in testosterone levels was observed at six months. While these levels remained within the normal range, it was already demonstrated that testosterone values typically return to normal within 12–18 months following m-TESE and eventually align with preoperative levels [18]. With extended follow-up, we will assess whether the same occurs after the l-TESE.
- Economic advantages: The adoption of surgical loupes offers significant economic advantages, particularly for healthcare facilities with limited resources. Operating microscopes, while providing high magnification and precise optical clarity, represent a major financial investment. These microscopes are equipped with advanced optical systems that allow for detailed magnification (20–36×), reducing eye strain for the surgeon during long procedures. However, the cost of these microscopes is elevated due to their complex design, which includes heavy optics supported by articulated arms that can hold the equipment in various positions, enhancing the precision but increasing the financial burden. In contrast, surgical loupes provide a more cost-effective alternative without substantially sacrificing the quality of the procedure. With magnifications ranging from 3.5× to 5×, loupes offer sufficient visualization to locate spermatogenic tubules, making them a viable option for sperm retrieval in patients with non-obstructive azoospermia (NOA). The simplicity of loupes, combined with their lightweight and portable design, makes them not only less expensive to purchase but also more practical for smaller clinics or resource-constrained centers that cannot afford the high costs associated with operating microscopes. By reducing the need for costly microsurgical equipment, l-TeSE makes the procedure more accessible, allowing facilities in low-resource settings to offer sperm retrieval services. This increased accessibility can improve the treatment outcomes for a broader population of NOA patients who may not otherwise have access to advanced reproductive care due to financial or infrastructural limitations. In this way, l-TeSE provides an economically feasible solution without compromising procedural success. These findings suggest that l-TeSE could be a safe and effective alternative to m-TeSE, particularly in resource-limited settings where access to advanced microsurgical training and equipment may be restricted.

None of our patients was a redo micro-TESE following an unsuccessful procedure even though this was not considered as an exclusion criterion for enrolling subjects in the analysis. Nevertheless, we do not believe that the l-TESE would have proven inferior to the M-TESE, even in this specific population. To date, the indications for performing a new surgical procedure following an unsuccessful M-TESE are still highly debated [29]. Redo m-TESE is typically performed as a last option before considering alternatives, like donor sperm or adoption. It is essential to have a thorough and transparent discussion with patients about the expected success rates of the procedure, as well as the potential risks involved, particularly the chances of developing testicular atrophy or hypogonadism. These complications can have significant long-term consequences on a patient’s reproductive health and hormonal balance, so they must be fully informed. Furthermore, it is of utmost importance to meticulously review all aspects of the previous surgical procedure, above all focusing on the result of the pathology analysis. This comprehensive approach ensures that both the patient and the medical team are aligned regarding the expectations and potential outcomes. According to a recent review [29], a redo micro-TESE can be advised or feasible in several situations. Specific factors, such as the histopathology of the initial biopsy, play a crucial role in determining the likelihood of success. For instance, men diagnosed with hypospermatogenesis, where sperm production is present but reduced, are more likely to benefit from a redo procedure. Patients with Klinefelter syndrome (KS) also show higher success rates in repeated procedures, with sperm retrieval occurring in up to 50% of cases in some studies. The timing between the first and second micro-TESE is another important consideration, with a recommended gap of 6–24 months. This allows for potential recovery or changes in the testicular environment that might enhance the chances of success. Additionally, addressing modifiable factors, like varicocele, or optimizing hormone levels through treatments, like human chorionic gonadotropin (hCG) or follicle-stimulating hormone (FSH), might further improve the outcomes in select patients. Though the sperm retrieval rates for the redo micro-TESE range between 10% and 21%, the variability in outcomes and lack of standardized treatment protocols make patient counseling crucial. Since none of these factors differed in our results, an l-TeSE could probably also be proposed for a redo TeSE.

Another scenario in which l-tese could play a role is Onco-TeSE. In patients with testicular cancer (TC), sperm cryopreservation is sometimes not feasible before surgery due to azoospermia, severe oligospermia, or challenges in collecting a semen sample. In such cases, performing surgical sperm retrieval (Onco-TESE) simultaneously with testicular surgery may enhance the reproductive potential of young individuals affected by TC, increasing their chances of preserving fertility. This must be obtained without delaying the treatment for TC [30]. The use of l-TESE could enable centers that cannot afford the operator microscope to perform onco-TESE more frequently. This could lead to an increase in fertility preservation among this population, without the need to risk delaying an orchiectomy in order to transfer the patient to a facility equipped for m-TESE.

Our study was not exempt from some limitations. With only 42 participants, this study may have lacked sufficient power to detect smaller differences or provide definitive conclusions. This limits the generalizability of the findings to a larger population of NOA patients. The six-month follow-up period may not be long enough to fully assess long-term outcomes, particularly regarding hormonal changes and potential postoperative complications, like hypogonadism. Moreover, this study excluded patients with genetic anomalies, such as Klinefelter syndrome or Y chromosome microdeletions. This limits the applicability of findings to a broader range of azoospermic patients, particularly those with complex genetic profiles. Finally, as this was a multi-center trial, differences in surgeon expertise and patient demographics across centers could introduce variability in results, potentially affecting the reproducibility of the study outcomes.

These factors should be considered when interpreting the results, and further studies with larger cohorts and longer follow-up periods are recommended to confirm these preliminary findings. However, we believe that this study benefitted from a controlled randomization, which enhanced the reliability of its findings and, to our knowledge, this was the first study that directly compared loupes and microscope use in the testicular sperm extraction procedure.

## 5. Conclusions

In conclusion, while m-TeSE remains the gold standard for sperm retrieval in NOA, l-TeSE offers a promising alternative with comparable efficacy, reduced operative time, and significant cost savings. Further randomized controlled trials are needed to confirm the preliminary findings of this study regarding retrieval rates, reduced operative times, and lower procedural costs.

## Figures and Tables

**Table 1:** Surgical and sperm analysis outcomes of patients who underwent micro-TeSE with surgical loupes or an operative microscope.

		Group A	Group B	
Variables	Total	Micro-TeSE	Surgical Loupes	*p*-Value
Number of testes, *n* (%)	84 (100)	42 (50)	42 (50)	
Positive sperm retrieval, *n* (%)	19 (22.6)	9 (21.4)	10 (23.8)	0.79
Histology, *n* (%)				0.73
Normal spermatogenesis	3 (3.6)	1 (2.4)	2 (4.8)	
Hypospermatogenesis	20 (23.8)	9 (21.4)	11 (26.2)	
Germ cell arrest	22 (26.2)	13 (31.0)	9 (21.4)	
Sertoli cell-only syndrome	39 (46.4)	19 (45.2)	20 (47.6)	
Johnsen score, *n* (IQR)	3.0 (2.0–3.4)	3.0 (2.0–3.2)	2.9 (2.0–4.0)	0.56
Sperm vials stored, *n* (IQR)	0 (0–0)	0 (0–0)	0 (0–1)	0.88
Operative time, min (IQR)	30 (26–33)	33 (31–38)	25 (24–27)	<0.01
Complications, *n* (%)	1 (1.2)	1 (2.4)	0 (0)	0.31

**Table 2:** Analysis of difference between preoperative and 6-month postoperative hormone values.

Variables	Preoperative	Postoperative	*p*-Value
Testosterone, ng/mL (IQR)	4.6 (3.5–5.6)	3.6 (3.2–6.1)	0.042
LH, mIU/mL (IQR)	7.4 (5.2–10.1)	8.9 (7.8–13.1)	0.845
FSH, mIU/mL (IQR)	16.5 (11.6–22.5)	16.9 (11.4–25.9)	0.945

## Data Availability

Additional data are available from the corresponding author upon reasonable request.
